# Accuracy of Contrast Extravasation on Computed Tomography for Diagnosing Severe Pelvic Hemorrhage in Pelvic Trauma Patients: A Meta-Analysis

**DOI:** 10.3390/medicina57010063

**Published:** 2021-01-12

**Authors:** Sung Nam Moon, Jung-Soo Pyo, Wu Seong Kang

**Affiliations:** 1 Department of Radiology, Wonkwang University Hospital, Iksan 54538, Korea; sungnam0422@gmail.com; 2 Department of Pathology, Eulji University School of Medicine, Uijeongbu Eulji University, Kyeonggi-do 11759, Korea; anapyojs@gmail.com; 3 Department of Trauma Surgery, Wonkwang University School of Medicine, Wonkwang University Hospital, Iksan 54538, Korea

**Keywords:** trauma, computed tomography, hemorrhage, angiography, pelvis

## Abstract

*Background and objective*: The early detection of underlying hemorrhage of pelvic trauma has been a critical issue. The aim of this study was to systematically determine the diagnostic accuracy of computed tomography (CT) for detecting severe pelvic hemorrhage. *Materials and Methods*: Relevant articles were obtained by searching PubMed, EMBASE, and Cochrane databases through 28 November 2020. Diagnostic test accuracy results were reviewed to obtain the sensitivity, specificity, diagnostic odds ratio, and summary receiver operating characteristic curve of CT for the diagnosis in pelvic trauma patients. The positive finding on CT was defined as the contrast extravasation. As the reference standard, severe pelvic hemorrhage was defined as an identification of bleeding at angiography or by direct inspection using laparotomy that required hemostasis by angioembolization or surgery. A subgroup analysis was performed according to the CT modality that is divided by the number of detector rows. *Result*: Thirteen eligible studies (29 subsets) were included in the present meta-analysis. Pooled sensitivity of CT was 0.786 [95% confidence interval (CI), 0.574–0.909], and pooled specificity was 0.944 (95% CI, 0.900–0.970). Pooled sensitivity of the 1–4 detector row group and 16–64 detector row group was 0.487 (95% CI, 0.215–0.767) and 0.915 (95% CI, 0.848–0.953), respectively. Pooled specificity of the 1–4 and 16–64 detector row groups was 0.956 (95% CI, 0.876–0.985) and 0.906 (95% CI, 0.828–0.951), respectively. *Conclusion*: Multi-detector CT with 16 or more detector rows has acceptable high sensitivity and specificity. Extravasation on CT indicates severe hemorrhage in patients with pelvic trauma.

## 1. Introduction

Pelvic fracture accompanying hemorrhage has been a large challenge for clinicians. Especially in hemodynamic instability due to pelvic ring injuries, mortality rates remain high (up to 35.7%) [1,2,3]. Bleeding is the most influential factor on the severity of pelvic injury. Therefore, early detection of underlying hemorrhage of pelvic trauma has been a critical issue. Despite well-experienced trauma surgeons, the surgeon’s gestalt is not sufficient to detect signs of catastrophe [4]. The previous prediction model for severe hemorrhage demonstrated predictors including mechanism of injury, systolic blood pressure, heart rate, hemoglobin, lactate, and focused abdominal sonography for trauma (FAST) [5]. However, there were no high-quality, evidence-based models. Contrast-enhanced computed tomography (CT) is a valuable diagnostic tool that reveals bleeding using contrast blush. Nevertheless, the role of CT is limited in hemodynamically unstable patients, because it can be a time-consuming process and delay resuscitation. However, both CT scanning and intervention are available simultaneously due to the recent revolutionary development of the hybrid-ER system and angio-CT suite in several trauma centers [6,7]. Moreover, since multi-detector CT has evolved, the diagnostic accuracy of CT has improved remarkably. Therefore, we conducted this systematic review and meta-analysis to determine the diagnostic test accuracy of CT for detecting severe pelvic hemorrhage. In contrast to the recent meta-analysis [8] that showed high sensitivity and specificity in 64-detector row CT, we intended to investigate if 16 or higher detector row CT might have a sufficient diagnostic accuracy.

## 2. Materials and Methods

### 2.1. Published Study Search and Selection Criteria

This study was performed in accordance with the Preferred Reporting Items for Systematic Reviews and Meta-Analysis of Diagnostic Test Accuracy (PRISMA-DTA) statement [9]. Relevant articles were obtained by searching PubMed, EMBASE, and Cochrane databases through 28 November 2020. These databases were searched using the following keywords: “(pelvic OR (pelvic fracture) OR (pelvic bone fracture)) AND (computed tomography) AND ((contrast extravasation) OR (arterial extravasation) OR (contrast blush) OR extravasation)).” In addition, we manually searched the reference lists of relevant articles. The titles and abstracts of all searched articles were screened for exclusion. Review articles and previous meta-analyses were also screened to obtain additional eligible studies. Search results were then reviewed, and articles were included if the study investigated the diagnostic accuracy of pelvic CT.

The inclusion criteria for diagnostic test accuracy (DTA) reviews were the following: (1) the study population included pelvic trauma patients; (2) as an index test, contrast-enhanced CT was performed. The positive finding on CT was defined as contrast extravasation; (3) as the reference standard, severe pelvic hemorrhage was defined as an identification of bleeding at angiography or by direct inspection using laparotomy that required hemostasis by angioembolization or surgery; (4) the purpose of the study was to evaluate the diagnostic accuracy of CT in pelvic trauma patients; (5) adequate information was provided to build a 2 × 2 table consisting of true positive (TP), false positive (FP), false negative (FN), and true negative (TN). Those articles that studied another disease, non-original articles, non-human study, pediatric study, or non-English-language publications were excluded.

### 2.2. Data Extraction

Data from all eligible studies were extracted by two investigators. Extracted data from each of the eligible studies included [10,11,12,13,14,15,16,17,18,19,20,21,22] the first author’s name, year of publication, study location, design, and period; the number of patients analyzed, type of rows of detector elements (1 to 64 detector row), and time from admission to angiography. In addition, the number of TP, FP, FN, and TN for contrast extravasation in diagnosing severe pelvic hemorrhage were collected.

### 2.3. Quality Assessment

All studies were independently reviewed by two investigators. Any disagreement concerning the study selection and data extraction were resolved by consensus. As recommended by the Cochrane Collaboration, to evaluate the risk of bias in DTA, the Quality Assessment of Diagnostic Accuracy Studies (QUADAS)-2 tool was used [23]. Disagreements were resolved by discussion with the third independent author. The QUADAS-2 assesses four domains for bias and applicability as follows: (1) patient selection: risk of bias is considered high if there is no consecutive patient enrollment and avoidance of case-control design or inappropriate exclusion; (2) index test: risk of bias is considered high if the index test results were interpreted without blinding to the reference standard; (3) reference standard: risk of bias is considered high if the reference standard could classify the target condition incorrectly; (4) flow and timing: risk of bias is considered high if not all patients were included with the same criteria or if there was an inappropriate time interval between the index test and reference standard.

### 2.4. Statistical Analysis

We investigated the contrast extravasation according to clinical characteristics from eligible studies and computed the point estimate to combine single descriptive statistics [24]. As the eligible studies used populations with heterogeneity, a random-effects model was more appropriate than a fixed-effects model. Heterogeneity between eligible studies was checked using probability statistics (*p*-value). To evaluate publication bias, Begg’s funnel plot and Egger’s test were conducted. If significant publication bias was found, the fail-safe N and trim-fill tests were also conducted to confirm the degree of publication bias. We conducted a bivariate diagnostic random effect meta-analysis that considers the joint distribution of sensitivity and specificity, allowing for a cross-study correlation. We calculated the pooled sensitivity, specificity, and diagnostic odds ratios (DOR) according to individual data collected from each eligible study in the various categories of comparison. As data were heterogeneous, accuracy data were pooled by fitting the summary receiver operating characteristics (SROC) curve (bivariate model) and measuring the area under the curve (AUC). An AUC close to 1 and 0.5 indicated a strong and poor test, respectively. Results with *p*-values <0.05 were considered statistically significant. Besides, we conducted additional meta-regression analysis by the location of eligible studies to find the possible cause of heterogeneity. All statistical analyses were conducted using a Comprehensive Meta-Analysis software package (Biostat, Englewood, NJ, USA) and R software (The R Foundation for Statistical Computing, version 3.6.0). In addition to base package of R, mada (version 0.5.8) and meta (version 4.10-0) packages were used.

## 3. Results

### 3.1. Selection and Characteristics

**Figure 1:**
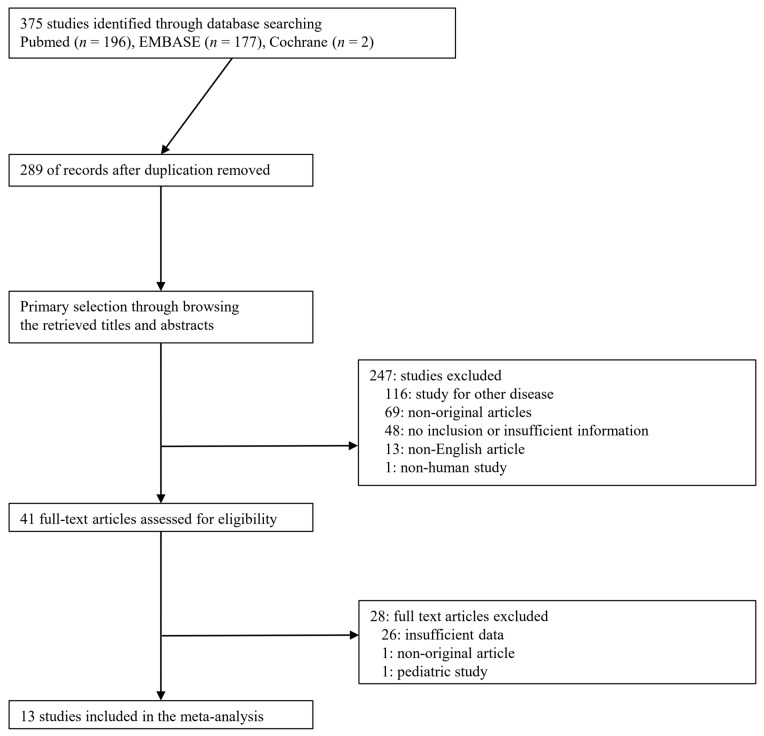
Flowchart summarizes literature and study selection.

A total of 375 studies were identified through database searching. Among the searched studies, 247 were excluded from title and abstract review because they were non-original (*n* = 69), studied other diseases (*n* = 116), non-human studies (*n* = 1), or were written in a non-English language (*n* = 13). After full-text review, 28 were excluded because they were insufficient data (*n* = 26), non-original article (*n* = 1), or pediatric study (*n* = 1). Finally, 13 studies (29 subsets) comprising 2642 patients were included in the present meta-analysis and DTA review (Figure 1), and detailed information about the eligible studies is shown in Table 1. To analyze for overall patients and subgroups, each study was investigated dividing into three subsets, such as overall, contrast extravasation, and no contrast extravasation.

### 3.2. Clinical Characteristics of Patients and Contrast Extravasation on CT

The estimated contrast extravasation positive rate of patients was 0.234 (95% CI, 0.15–0.334; heterogeneity test, *p* < 0.001; Egger’s test, *p* = 0.529; 13 studies and 15 subsets) [10,11,12,13,14,15,16,17,18,19,20,21,22]. The estimated points of contrast clinical characteristics according to contrast extravasation on CT are summarized in Table 2 [10,14,15,16,19,20,21]. Age, injury severity score, and mortality rate were significantly higher in the positive-extravasation group.

### 3.3. DTA Review

**Figure 2:**
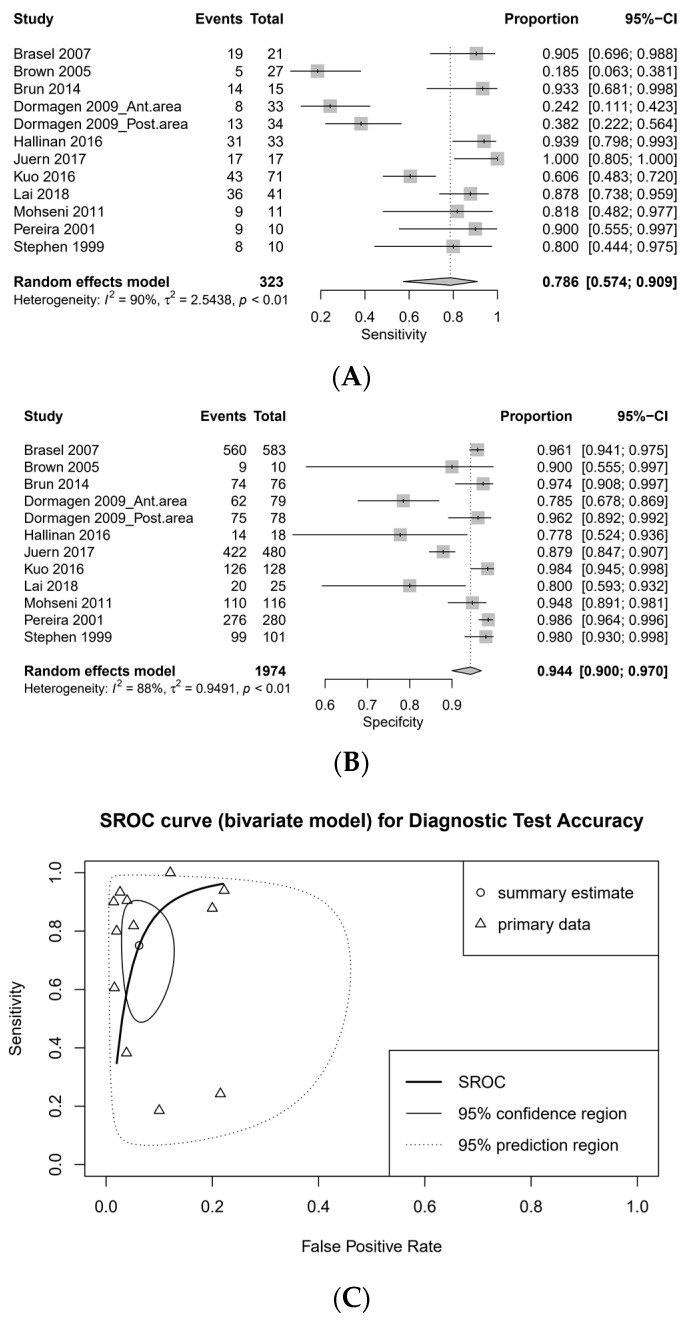
Forest plots for sensitivity (**A**) and specificity (**B**) and summary receiver operating characteristics (SROC) curve (**C**).

The pooled sensitivity of CT was 0.786 (95% CI, 0.574–0.909, I2 = 90%), and the pooled specificity was 0.944 (95% CI, 0.900–0.970, I2 = 88%; Figure 2). The diagnostic odds ratio (DOR) of CT was 53.545 (95% CI, 14.728–194.665) and the area under curve (AUC) on summary receiver operating characteristics (SROC) was 0.994. There was no threshold effect by calculating Spearman’s rank correlation coefficient (*r* = −0.105, *p* = 0.750).

### 3.4. Sensitivity Analysis and Subgroup Analysis

**Figure 3:**
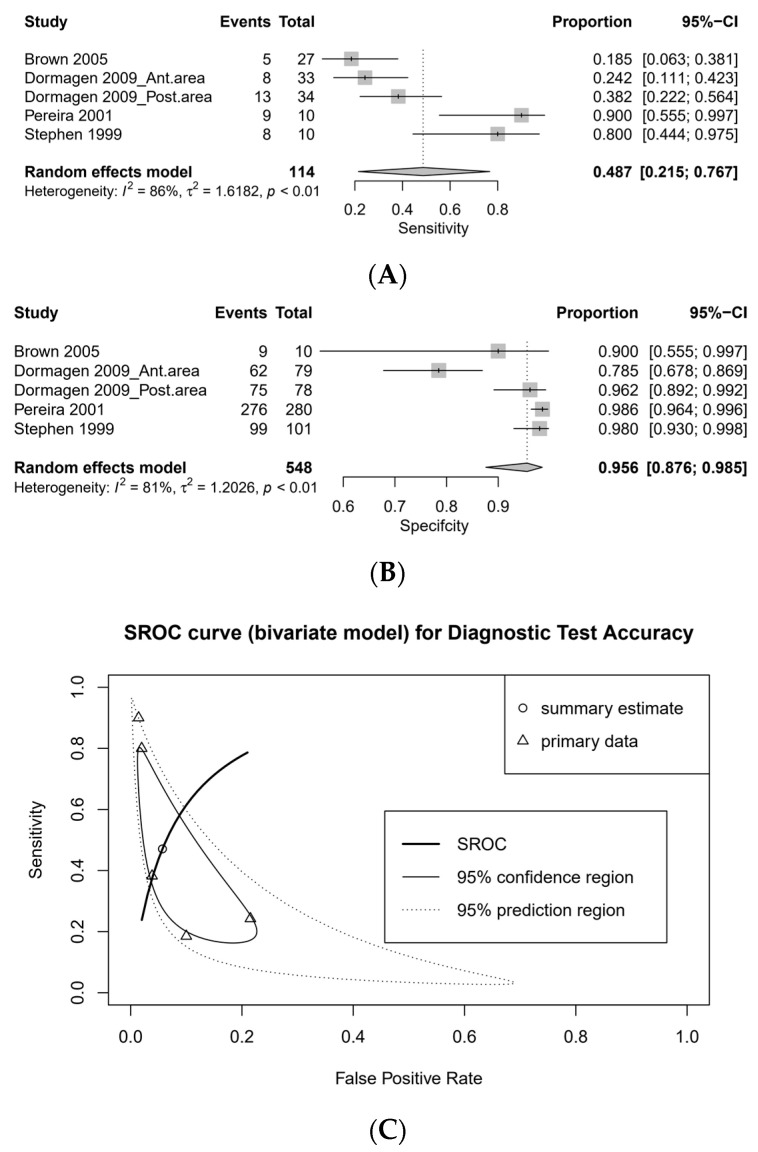
One to four detector row: forest plots for sensitivity (**A**), specificity (**B**), and SROC curve (**C**).

Two studies did not describe whether multi-detector CT was used or not (Table 1). For sensitivity analysis, studies with non-descriptive CT modalities or low-quality CT (1–4 detector row) were deleted (pooled sensitivity, 0.786 [95% CI, 0.574–0.909] vs. 0.915 [95% CI, 0.848–0.953]; pooled specificity, 0.944 [95% CI, 0.900–0.970] vs. 0.906 [0.828–0.951]; Figure 3).

**Figure 4:**
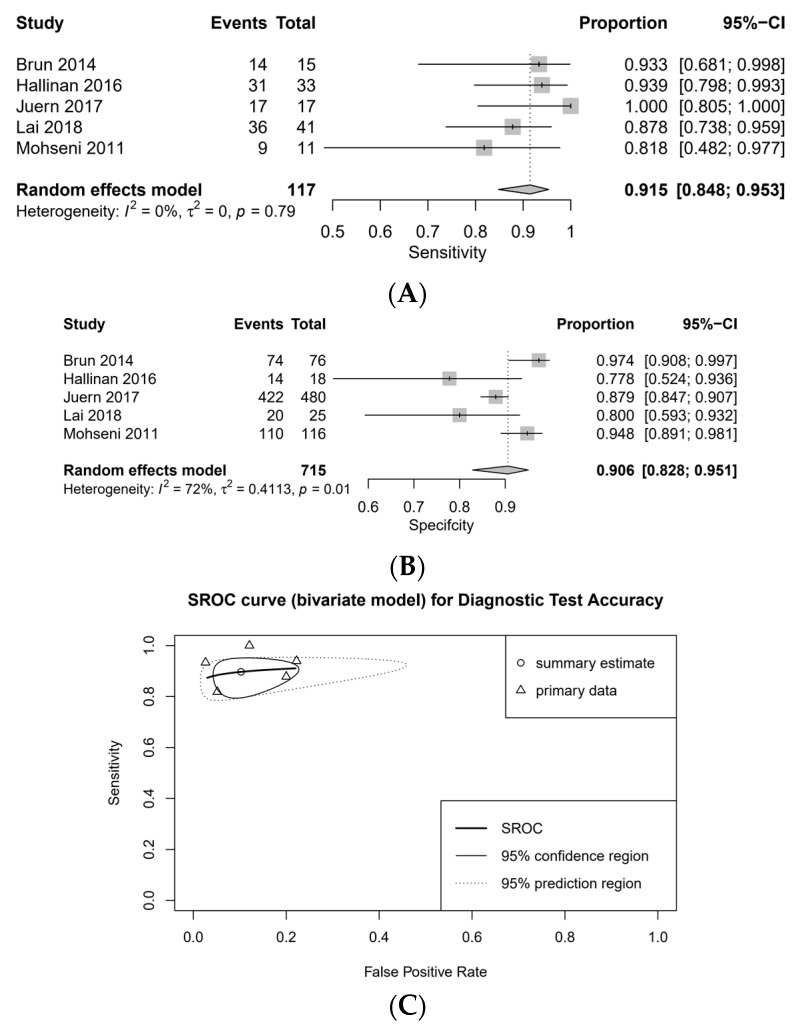
16–64 detector row: forest plots for sensitivity (**A**), specificity (**B**), and SROC curve (**C**).

Subgroup analysis was performed according to the CT modality that is divided by the number of detector rows that affects the quality of the image (Figure 3 and Figure 4). The pooled sensitivity of the 1–4 detector row group was low (0.487 [95% CI, 0.215–0.767, I2 = 86%]), while the pooled sensitivity of the 16–64 detector row group was high (0.915 [95% CI, 0.848–0.953, I2 = 0%]). The pooled specificity of the 1–4 and 16–64 detector row groups was high (0.956 [95% CI, 0.876–0.985, I2 = 81%] and 0.906 [95% CI, 0.828–0.951, I2 = 72%], respectively). The DOR was 19.582 (95% CI, 1.909–200.872) in 1–4 detector row group and 76.178 (95% CI, 29.261–198.320) in 16–64 detector row group, respectively. The AUC on SROC was 0.885 in 1–4 detector row group and 0.915 in 16–64 detector row group, respectively. There were no significant differences in the diagnostic accuracy of contrast extravasation on CT among the location of studies (Table 3).

### 3.5. Quality Assessment

**Figure 5:**
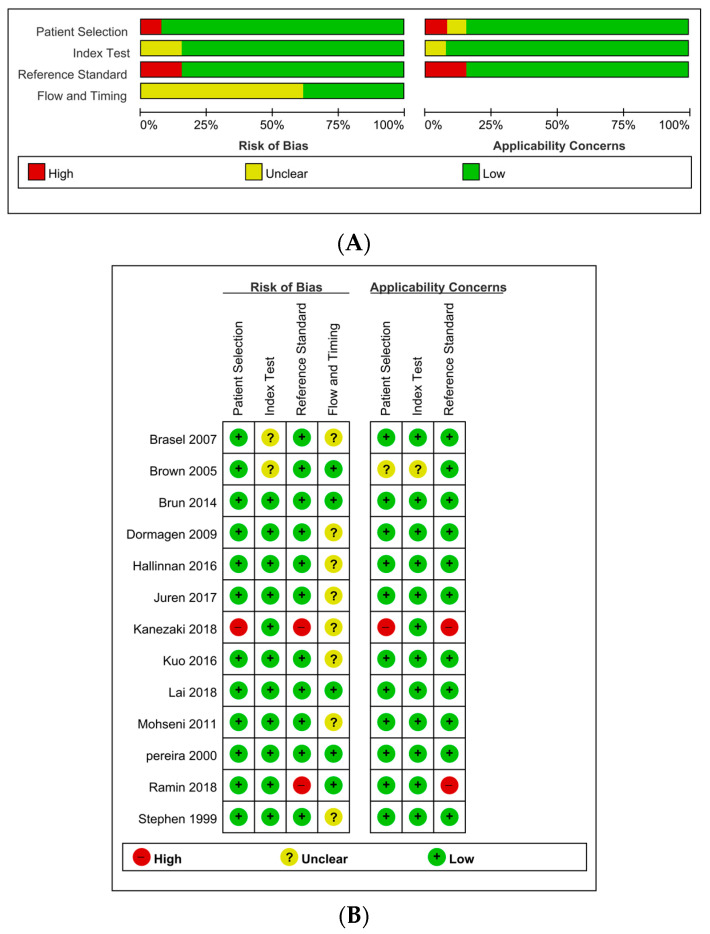
Risk of bias and applicability concerns graph (**A**) and summary (**B**): review authors’ judgements about each domain presented as percentages across included studies.

The details of the quality assessment are described in Figure 5. In terms of test flow and timing, the risk of bias was unclear in eight studies (61.5%). Only seven studies (53.8%) showed the time from admission to hemostasis (angioembolization or surgery). Moreover, several studies had a long duration from admission to identify the bleeding via angiography or surgery (7–24 h) [11,14,22]. Two studies had a significantly high risk of bias in terms of reference standards [16,21]. These studies include resuscitative endovascular balloon occlusion of the aorta (REBOA) and external pelvic fixation that are not amenable to directly identify the bleeding focus. Indeed, REBOA needs consecutive surgical or angiographic hemorrhagic control. We decided that these modalities are not suitable for the reference standard. Therefore, we did not calculate the sensitivity and specificity in these studies. In contrast to these two studies, one eligible study [19] included a bilateral internal iliac artery ligation through laparotomy that was amenable to directly identify the presence of bleeding.

### 3.6. Publication Bias

To assess publication bias, Begg’s funnel plot and Egger’s test were preferentially conducted. In the estimated mortality rate according to contrast extravasation, there was a significant publication bias (*p* = 0.078; Table 2). According to contrast extravasation, there were significant biases (overall, *p* = 0.010; positive contrast extravasation, *p* = 0.003; negative contrast extravasation, *p* = 0.016; Table 3). To define the degree of publication bias, the fail-safe N and trim and fill tests were conducted as secondary assessments, and no significant publication bias was found (Table 3). In assessing other subgroups, no significant publication bias emerged.

## 4. Discussion

Our results suggest that contrast extravasation on the CT in pelvic trauma patients showed acceptable diagnostic accuracy, especially in multi-detector CT. In several early conducted studies, 1–4 detector row CT showed low sensitivity and limited diagnostic value. However, recent multi-detector row CT (16–64 detector row) showed sufficient sensitivity and specificity. In a recent meta-analysis, subgroup analysis showed pooled sensitivity and specificity of 94 and 89% for 64-detector row CT [8]. The present study demonstrated that even the 16–64 detector row CT showed sufficient sensitivity (91.5%) and specificity (90.6%). Furthermore, we noted that the pooled estimates of age, ISS, and mortality rate were higher in contrast extravasation group. Whereas, we defined more strict criteria regarding the reference test that comprised angiography and surgical diagnosis (direct inspection by a surgeon), the previous meta-analysis [8] used ambiguous reference tests such as external fixation or preperitoneal pelvic packing that are not able to identify the real arterial hemorrhage directly. We also defined more strict criteria regarding positive angiography, whereas the previous meta-analysis [8] comprised decision of embolization. This contributed to a smaller number of eligible studies in our analysis. A rigorous and explicit definition of reference standard should be needed to reduce the heterogeneity and the risk of bias in diagnostic test accuracy review [25]. Nevertheless, there was heterogeneity and substantial risk of bias in terms of timing of index test and reference standard in the present study.

In the present study, we focused on extravasation on CT that indicates bleeding from the injured vessel. In contrast to our results, in a retrospective review including 162 pelvic ring fracture patients, pelvic blushes with stable vital signs were successfully managed without surgical or radiological hemorrhagic control [26]. Whereas the role of CT is limited in hemodynamically unstable patients [27], the pelvic angiography/angioembolization, in patients with arterial contrast extravasation on CT, may have a benefit regardless of hemodynamic status [28] Clinically, false-negatives for pelvic hemorrhage are more dangerous than false-positives, because a failure to recognize the underlying bleeding can lead to catastrophe. The false-negative rates of the present analysis were 51.3% in 1–4 detector row and 8.5% in 16–64 detector row, respectively. In a recent retrospective review using modern 64-detector row CT scanners [15], 100% negative predictive value was reported, whereas another previous study [17] reported 28 positive angiography patients among 154 negative contrast blushes on CT (18.2%). However, the detector row type of the CT scanner was not described in that study. Currently, the absence of contrast blush cannot exclude active pelvic bleeding [28].

In our analysis, we found that multi-detector CT has sufficient sensitivity and specificity for detecting severe hemorrhage. In a recent meta-analysis, no serious pelvic injuries were found when physical examination findings were normal [29]. A portable pelvic radiograph is not an effective diagnostic tool to detect pelvic fractures, because it often failed to detect sacral and iliac fractures [30]. Focused abdominal sonography for trauma (FAST) is a useful option for early detection of intraperitoneal fluid (hemorrhage), but it is limited to the detection of retroperitoneal hemorrhages [27,31]. The previously reported diagnostic accuracy of FAST for hemorrhagic pelvic fracture was poor (26% sensitivity, 96% specificity, 85% positive predictive value, and 63% negative predictive value, respectively) [32]. Nevertheless, many clinicians still prefer FAST to CT in unstable patients due to the limitation of CT [33]. A recent randomized trial [34] regarding whole-body CT (WBCT) for severe trauma patients did not reveal a significant difference in terms of in-hospital mortality, whereas a meta-analysis [35] revealed a favorable outcome (pooled odds ratio in WBCT group for 24 h morality, 0.72 [95% CI, 0.66–0.79]). Therefore, CT remains a controversial initial diagnostic tool in unstable patients. The recent management algorithm of pelvic fracture depends on hemodynamic instability [27]. Pelvic injuries range from low-energy simple fractures to high-energy unstable patterns that can lead to severe lethal hemorrhages. Thus, emergent resuscitation should be a priority over CT scanning in unstable patients. Recently, various treatment modalities, including hemostatic resuscitation guided by viscoelastic testing, REBOA, preperitoneal pelvic packing, and external fixation, are performed without CT scanning, according to hemodynamic instability, although the exact role of REBOA is not determined yet [2,27]. For CT scanning, the patient should be transferred to the CT room from the resuscitation or emergency room, where it will take tens of minutes to complete the examination. Since this is a time-consuming procedure and the patient’s instability can worsen, ultrasonography or portable plain pelvic radiography is preferred over CT scanning [36].

The present study has several limitations. First, all eligible studies were retrospective, thus selection bias could not be avoided. Second, the present study has limitations concerning the heterogeneity of reported data. Although the statistical heterogeneity was substantially high in all eligible studies, the studies using 16–64 detector row CT showed low heterogeneity after subgroup analysis as well as sufficient summary estimates of sensitivity. There was substantial heterogeneity across the studies regarding the duration from admission to reference standard. The description of the timing of CT scanning was absent in all studies. Thus, the appropriate interval between index test and reference standard is unclear. This suggests the need for future application and investigation of the hybrid–ER system. Third, the quality of CT modalities varied across the studies. However, by performing subgroup analysis, we revealed that modern multi-detector CT has acceptable diagnostic accuracy. Fourth, the publication-bias-adjusted pooled estimates after using the trim-and-fill method were not significantly different. However, the small number of studies in the present meta-analysis could provide little precision [37]. Finally, we could not separate the datasets according to the hemodynamic status. Thus, a further study addressing the diagnostic value of CT alone, regardless of vital signs, is warranted.

## 5. Conclusions

Our meta-analysis demonstrated that modern multi-detector CT, with 16 or more detector rows, has acceptable high sensitivity and specificity, whereas 1–4 detector row CT has limitations in diagnosis. We found that even the CT with 16 detector rows has sufficient accuracy compared to the previous meta-analysis [8]. Extravasation on CT indicates severe hemorrhage in pelvic fracture patients.

## Figures and Tables

**Table 1:** Main characteristics of the eligible studies.

Study	Location	Study Design	Study Period	Trauma Type	CT Modality (Detector Row)	Contrast Agent Infusion Amount, Rate	Scan Time after Contrast Agent Infusion	Reference Standard	Hemostatic Modality	Time from Admission to Hemostasis	Subgroup	Number of Patients		
												Total	CE (+)	CE (−)
Brasel 2007	USA, single level 1 center	Re	1998–2005	ND	4, 8, 16	150 mL, 5 mL/s	60 s	Angiography	AE	ND		604	42	562
Brown 2005	USA, single level 1 center	Re	2001–2003	ND	1	350 mL, 2 mL/s	ND	Angiography	AE	7 ± 4 h		37	6	31
Brun 2014	France, single level 1 center	Re	2004–2008	ND	40, 64	ND	ND	Angiography	AE	120 (77–191) min, median(IQR)	Stabilized patients	95	16	75
											Unstabilized patients			
Dormagen 2009	Norway, single center	Re	1995–2007	ND	1,2,4	100–150 mL, 2–5 mL/s	70 s	Angiography	AE	ND	Anterior area	112	25	87
											Arterial blush			
											Posterior area	112	34	78
											Arterial blush			
Hallinan 2016	Singapore, single center	Re	2004–2012	Blunt	16 or 64	100 mL, 3 mL/s	70 s	Angiography	AE	within 24 h		51	35	16
Juern 2017	USA, single level 1 center	Re	2009–2014	Blunt	64	125 mL, 2.5 mL/s	70 s	Angiography	AE	ND		497	75	422
Kanezaki 2016	Japan, single center	Re	2012–2015	ND	ND	ND	ND	Angiography	AE	ND	Geriatric group	15	10	5
								Surgery	IABO					
									Pelvic fixation					
									Surgical hemostasis					
											Younger group	13	6	7
Kuo 2016	Taiwan, single center	Re	2005–2012	ND	ND	ND	ND	Angiography	AE	ND		201	47	154
Lai 2018	Taiwan, single center	Re	2012–2014	ND	64	100 mL, 3 mL/s	70 s	Angiography	AE	3.2 h, mean		66	41	25
Mohseni 2011	USA, single level 1 center	Re	2008–2010	Blunt	64	100 mL, 3 mL/s	ND	Angiography	AE	ND		127	15	112
								Surgery	BILA					
									PP					
Pereira 2001	USA, single level 1 center	Re	1994–1999	ND	1	150 mL, 2 mL/s	60 s	Angiography	AE	3.3 ± 0.4 h		290	13	277
Ramin 2018	France, single level 1 center	Re	2010–2015	ND	64	140 mL, 4 mL/s	70–80 s	Angiography	AE	120 (90–560) min, median (IQR)		311	94	217
								Surgery	PP					
									Pelvic fixation					
Stephen 1999	Canada, two level 1 centers	Re	1995–1996	ND	1	100 mL, 2 mL/s	65 s	Angiography	AE	11 (3–49) hours, median (range)		111	10	101

CT, computed tomography; CE, contrast extravasation; Re, retrospective; ND, no description; AE, angioembolization; IABO, intra-aortic balloon occlusion; PP, pelvic packing; IQR, interquartile range; SD, standard deviation; BILA, bilateral ligation of iliac arteries.

**Table 2:** Estimate points of clinical characteristics according to contrast extravasation on computed tomography in patients with pelvic fracture.

	Number of Subsets	Fixed Effect (95% CI)	Heterogeneity Test (*p*-Value)	Random Effect (95% CI)	Egger’s Test (*p*-Value)
Age, mean					
CE (+)	5	49.431 (47.195, 51.667)	0.326	49.251 (46.761, 51.742)	0.361
CE (−)	5	39.032 (38.904, 39.161)	<0.001	40.185 (38.241, 42.129)	0.332
Sex, male rate					
CE (+)	5	0.623 (0.559, 0.683)	0.074	0.615 (0.511, 0.710)	0.660
CE (−)	5	0.601 (0.577, 0.625)	0.279	0.603 (0.575, 0.631)	0.110
ISS, mean					
CE (+)	4	30.762 (28.834, 32.690)	<0.001	29.536 (24.215, 34.857)	0.537
CE (−)	4	18.210 (18.117, 18.304)	<0.001	19.184 (17.707, 20.660)	0.413
Mortality, rate					
CE (+)	7	0.163 (0.125, 0.208)	0.682	0.163 (0.125, 0.208)	0.078
CE (−)	5	0.057 (0.045, 0.072)	0.015	0.048 (0.029, 0.078)	0.046

CI, confidence interval; CE, contrast extravasation; ISS, injury severity score.

**Table 3:** Meta-regression of factors associated with diagnostic accuracy of contrast extravasation on computed tomography in patients with pelvic fracture.

	Number	Fixed Effect	Heterogeneity Test	Random Effect	Egger’s	Trim and Fill Test	
	of	[95% CI]	[*p*-Value]	[95% CI]	Test		* Meta-Regression Test [*p*-Value]
	Subsets				[*p*-Value]		
Overall patients	24	0.730 (0.694, 0.762]	<0.001	0.861 (0.766, 0.922]	0.01	0.884 (0.779, 0.943]	
America	12	0.602 (0.532, 0.669]	<0.001	0.896 (0.693, 0.970]	<0.001	0.862 (0.629, 0.958]	
Asia	6	0.845 (0.799, 0.882]	0.335	0.852 (0.798, 0.893]	0.102	-	0.948
Europe	6	0.728 (0.667, 0.782]	<0.001	0.777 (0.597, 0.891]	0.526	-	0.448
1–4 detector row	10	0.707 (0.650, 0.758]	<0.001	0.793 (0.616, 0.901]	0.345	-	
America	6	0.715 (0.600, 0.807]	<0.001	0.881 (0.514, 0.981]	0.052	-	
Europe	4	0.704 (0.638, 0.763]	<0.001	0.672 (0.475, 0.823]	0.648	-	0.227
16–64 detector row	10	0.693 (0.620, 0.759]	<0.001	0.897 (0.702, 0.970]	<0.001	0.933 (0.682, 0.989]	
America	4	0..455 (0.348, 0.567]	<0.001	0.904 (0.360, 0.994]	0.047	0.904 (0.360, 0.994]	
Asia	4	0.860 (0.784, 0.913]	0.785	0.860 (0.784, 0.913]	0.799	-	0.886
Europe	2	0.942 (0.834, 0.982]	0.061	0.954 (0.674, 0.995]	-	-	0.563
CE positive patients	12	0.566 (0.499, 0.632]	<0.001	0.723 (0.533, 0.856]	0.003	0.655 (0.470. 0.802]	
1–4 detector row	5	0.600 (0.465, 0.720]	0.01	0.683 (0.427, 0.861]	0.079	-	
16-64 detector row	5	0.544 (0.559, 0.636]	<0.001	0.729 (0.355, 0.929]	0.057	-	
CE negative patients	12	0.814 (0.714, 0.845]	<0.001	0.937 (0.859, 0.974]	0.016	0.867 (0.726, 0.941]	
1–4 detector row	5	0.736 (0.674, 0.789]	<0.001	0.867 (0.634, 0.961]	0.343	-	
16–64 detector row	5	0.934 (0.882, 0.964]	<0.001	0.968 (0.855, 0.994]	0.052	-	

CI, confidence interval; *, compared to studies from America; CE, contrast extravasation.

## Data Availability

Data is contained within the article.
